# Light Limitation of Poleward Coral Reef Expansion During Past Warm Climates

**DOI:** 10.1029/2024GL111757

**Published:** 2024-11-21

**Authors:** A. L. Kruijt, T. Brachert, A. Sluijs, J. J. Middelburg

**Affiliations:** ^1^ Department of Earth Sciences Faculty of Geosciences Utrecht University Utrecht The Netherlands; ^2^ Institute for Earth System Science and Remote Sensing University of Leipzig Leipzig Germany

**Keywords:** biogeography, corals, light limitation, climate, eocene, habitat modeling

## Abstract

The latitudinal range of modern shallow‐water tropical corals is controlled by temperature, and presently limited to waters warmer than 16–18°C year‐round. However, even during Cenozoic climates with such temperatures in polar regions, coral reefs are not found beyond >50° latitude. Here, we test the hypothesis that daily available solar radiation limited poleward expansion of coral reefs during warm climates, using a new box model of shallow marine coral calcification. Our results show that calcification rates start to decline beyond 40° latitude and drop severely beyond 50° latitude, due to decreasing winter light intensity and day length, irrespective of aragonite saturation. This suggests that light ultimately prohibits further poleward expansion in warm climates. In addition, fossil coral reef distribution is not a robust proxy for water temperatures and poleward expansion of reefs beyond 50° latitude is not an expected carbon cycle feedback of climate warming.

## Key Points

- The uppermost latitudinal limit of tropical coral reefs was limited by winter daily available radiation during past warm climates
- Fossil coral reef distribution is not a robust proxy for water temperatures
- For the correct interpretation of coral reef records, coral‐light relationships need to be taken into consideration

## 1 Introduction

### 1.1 Environmental Drivers of Coral Reef Distribution

Shallow‐water tropical corals (from now on “corals”) are found roughly between 30°N and 30°S. Their latitudinal distribution is typically considered to be temperature controlled and expected to extend or shift poleward with rising water temperatures, as also occurred in warmer‐than‐present periods in the geological past (Jones et al., 2022; Schlager, 1992). Migration to higher latitudes may help coral ecosystems survive modern global warming, but it is important to understand how far these habitats can extend (Hoegh‐Guldberg et al., 2017; Sommer et al., 2018). Not only temperature, but many other environmental drivers play a role in the distribution of corals. Kleypas et al. (1999) showed that aragonite saturation state, light availability and temperature together determine the extent of present‐day coral reef habitats and that corals living at their environmental limits are often limited by a combination of drivers that are hard to separate. Light availability is widely recognized to determine the maximum depth at which corals and their phototrophic symbionts can thrive, as well as species richness along depth gradients (e.g., Heiss, 1995; López‐Londoño et al., 2022). Muir et al. (2015) highlighted the importance of winter irradiance on latitudinal gradients in species richness. They analyzed a global data set of staghorn corals and showed that corals are confined to shallower depths at higher latitudes. Based on these results they hypothesized that the latitudinal attenuation of winter daily irradiance levels would ultimately pose a constraint on latitudinal coral range expansion in a warming ocean. Their results have however been contested by others (Madin et al., 2016), who for example, point out that Muir's study does not explain latitudinal coral expansion during past warm climates as observed in the geological record. Many researchers have pointed at the latitudinal gradient in aragonite saturation state as a potential limitation to coral reef expansion (e.g., Abrego et al., 2021; Buddemeier & Smith, 1999; Kleypas et al., 1999). Corals presently live at low latitudes, in warm waters highly saturated in aragonite. Saturation state in the modern ocean is lower at high latitudes due to lower water temperatures and decreases globally as CO_2_ levels increase (Heinze et al., 2021). Poleward expansion of coral reefs could therefore become problematic due to lower saturation levels (Huang et al., 2024).

### 1.2 Cenozoic Coral Reef Distributions

**Figure 1:**
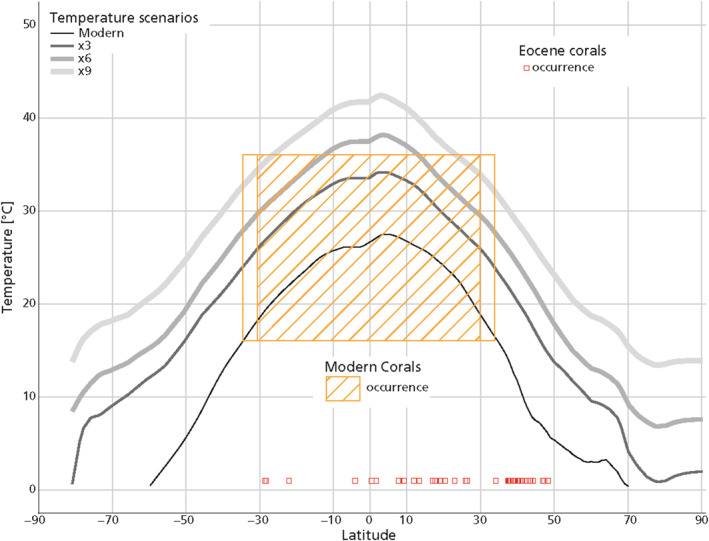
Meridional coldest month mean sea surface temperature gradients in the modern and in three Eocene simulations (3x, 6x, and x9 modern CO_2_; Community Earth System Model 1.2; Zhu et al., 2019). Orange box indicates modern latitudinal coral range, with most corals occurring within the hatched area; red squares indicate latitudes of Eocene coral fossil reefs (PARED database, Kiessling & Krause, 2022).

The latitudinal range fluctuations of coral reef patterns throughout the Cenozoic have been shown to result from many interacting factors, and commonly do not cross‐correlate with paleoclimatic change (Flügel & Flügel‐Kahler, 1992; Perrin & Kiessling, 2010). For instance, Kiessling et al. (2012) showed that interglacial warming caused not only poleward shifts but also loss of equatorial coral diversity. However, one striking feature of these coral reef patterns throughout the Cenozoic, is that coral reefs are never recorded beyond 50° paleolatitude (Perrin & Kiessling, 2010). This is particularly unexpected for the Early and Middle Eocene, when surface water temperatures at mid and high latitudes were 10° to 20°warmer than today and would still have been unlimiting at 50°N (e.g., Hollis et al., 2019) and temporally even in the Arctic (e.g., Sluijs et al., 2006, 2020; Suan et al., 2017; Willard et al., 2019). Since corals reefs did not extend as far poleward as their present‐day temperature tolerance would allow (Figure 1; Perrin & Kiessling, 2010; Brachert et al., 2022), another factor must have limited growth at mid to high latitudes. Because temperature did not limit further poleward expansion (Figure 1), we explore the hypothesis (Muir et al., 2015) that critically low light levels limited the occurrence of corals to <50°N during the Eocene. We test this hypothesis using a new simple model for coral calcification that is based on the premise that other environmental drivers like nutrient regimens, turbidity and aragonite saturation state were not limiting at these latitudes. Surface oceans were supersaturated for aragonite over most of the Cenozoic (Boudreau et al., 2019) and the warm conditions must have further boosted saturation. Although high *p*CO_2_ might have caused lower ocean water pH, there is no evidence for critically low saturation states during this time even in high‐latitude surface waters (Kump et al., 2009). For testing this specific hypothesis, we therefore do not include a latitudinally varying aragonite saturation state in our model, but study the interplay between temperature and light and how they determine the latitudinal range of corals.

## 2 Materials and Methods

Our simple box model reflects an arbitrary volume of water in which coral calcification can occur. The calcification rate (G) depends on the latitudinally changing light‐ and temperature regime as follows:

(1) $$G=\text{Gmax}*f_{\text{light}}\left(E_{\text{surface}}\;(\text{lat},z\right)*\;f_{\text{darkdays}}\left(\text{lat}\right)*f_{\text{temp}}\left(\text{Temperature}\;\left(\text{lat}\right)\right)$$

where Gmax refers to unlimited, or “100%” calcification potential, and *f*_light_, *f*_darkdays_ and *f*_temp_ are limitation functions.

### 2.1 Light Limitation

The function *f*_light_ takes available light (*E*_*z*_) as its argument, which is a function of latitude (lat) and water depth (z). The available light at the surface of the earth (*E*_surface_) for each day of the year at each latitude is determined by the declination of the earth and the sun hour angle (ω_s_) following Berger (1978; Equations S1–S9 in (Supporting Information S1). We note that this varies within certain limits with Milankovitch cyclicity through geological time but for simplicity we assume the modern orbit, which underestimates light availability for orbital extremes, for a first order assessment.

Light availability limits coral growth to shallow depth in turbid waters but deeper habitats are possible in clear water (Grigg & Epp, 1989; Jarrett et al., 2005). The available light (*E*_*z*_) is a function of the light attenuation coefficient (kpar) and water depth (z) according to the Lambert‐Beer equation:

(2) $$E_{z}=E_{\text{surface}}*e^{-\text{kpar}*z}$$

The unitless product kpar*z is critical for light available to corals: we varied it between 0.15 and 3.00, corresponding to kpar values between 0.01 and 0.20 m^−1^ for corals at 15 m water depth, that is, photic zones depths (4.6/kpar) between 23 and 460 m. For corals living at 3 m, kpar and photic zones would range from 0.05 to 1 m^−1^ and 4.6 to 92 m. Light becomes a limiting factor to coral calcification when the available light falls below the saturating light intensity (*E*_*k*_). Following previous work (Boscher & Schlager, 1992; Jassby & Platt, 1976; Kleypas, 1997) this relationship between coral growth and light is calculated with a hyperbolic tangent function:

(3) $$f_{\text{light}}=\tanh\left(\frac{E_{z}}{E_{k}}\right)$$

The saturating light intensity *E*_*k*_ varies greatly among coral species. Boscher and Schlager (1992) report values of between 50 and 450 μmol m^−2^ s^−1^ and Kleypas (1997) uses different light saturation intensities of between 50 and 300 μmol m^−2^ s^−1^ in model simulations of global reef distribution. We followed this approach and tested the sensitivity of our model to *E*_*k*_ by using *E*_*k*_ values of 50, 100, 150, 200, and 300 μmol m^−2^ s^−1^. For the final simulations including both temperature‐ and light limitation, an *E*_*k*_ of 50 μmol m^−2^ s^−1^ and a combination of z*kpar = 0.75 is used, resembling a coral reef at 15m in clear water (photic zone of 92 m). We further include a “dark‐day tolerance” determined by *E*_lim_, representing the threshold value that daily light levels must reach for a coral to calcify. As with *E*_*k*_, *E*_lim_ varies among species and its value is not well established (Gattuso et al., 2006; Kleypas, 1997). In our simulations we test a range of values from 20 to 400 μmol m^−2^ s^−1^. Daily light levels at the surface (*E*_surface_) for each day of the year at each latitude are compared to *E*_lim_, leading to a total number of “dark days” per year at each latitude. Light levels at the surface are used here rather than *E*_*z*_, to exclude the possibility that a coral population might have migrated upward to receive more daylight and escape from the “darkness” at depth. A simple dark‐day tolerance function is then used to test the latitudinal limits to coral occurrence based on different dark‐day tolerances.

$$f_{\text{darkdays}}\left(\text{number}\;\text{of}\;\text{dark}\;\text{days}>\text{tolerance}\right)=0$$

(4) $$f_{\text{darkdays}\;}\left(\text{number}\;\text{of}\;\text{dark}\;\text{days}<\text{tolerance}\right)=1$$

### 2.2 Temperature Limitation

For temperature (*f*_temp_), we applied three different Eocene coldest month mean and highest month mean sea surface temperature distributions based on the results of Earth System Model simulations for the Eocene with x3, x6 and x9 pre‐industrial CO_2_ conditions (Zhu et al., 2019). These simulations show reasonable correspondence to paleotemperature reconstructions, the x6 CO_2_ showing the better fit with the available proxy data (Lunt et al., 2021). These simulations in general somewhat underestimate polar amplification of greenhouse warming (Fokkema et al., 2024; Gaskell et al., 2022), which is why we include the x9 CO_2_ simulation as a plausible scenario for high latitude temperatures. We also applied a modern‐day temperature distribution (Locarnini et al., 2019) to test the model for the present day.

Reef corals are found in waters of roughly between 23 and 30°C (e.g., Lough & Cantin, 2014) with extremes of 16–36°C reported for some species. (e.g., D’Angelo et al., 2015; Tuckett & Wernberg, 2018). For simplicity, we did not discriminate between different coral species and their temperature optima and tolerance curves but assume that a coral either does or does not calcify at a certain temperature (Equation 5). We allow for calcification only within the 16–36°C temperature envelope. This allows for coral growth at relatively high and low temperatures, which implies a conservative temperature limitation for latitudinal expansion.

$$f_{\text{temp}}\left(\;\text{Temperatur}e_{\text{winter}}<16\;\right)=0$$

$$f_{\text{temp}}\left(16\leq Temperature_{\text{winter}}\;\&\;Temperature_{\text{summer}}\leq 36\right)=1$$

(5) $$f_{\text{temp}}\left(\text{Temperatur}e_{\text{summer}}>36\right)=0$$

We computed daily and yearly calcification rates at latitudes 0–90°, at one degree resolution. The sensitivity of the light‐limitation to variations in the value of *E*_*z*_ and *E*_*k*_ was tested using a latitudinally constant, unlimiting temperature distribution of 26°C. The interplay between light and temperature was then studied by simulating calcification for the three Eocene temperature scenarios and the present day temperature distribution, under a fixed value of z*kpar (15 m × 0.05 m^−1^) and *E*_*k*_ (50 μmol m^−2^ s^−1^). *E*_lim_ was varied between 20 and 400 μmol m^−2^ s^−1^ and the tolerance to dark days was varied between 1 and 365 days, in order to determine which combination of these parameters best represents the Eocene coral reef record.

## 3 Results

**Figure 2:**
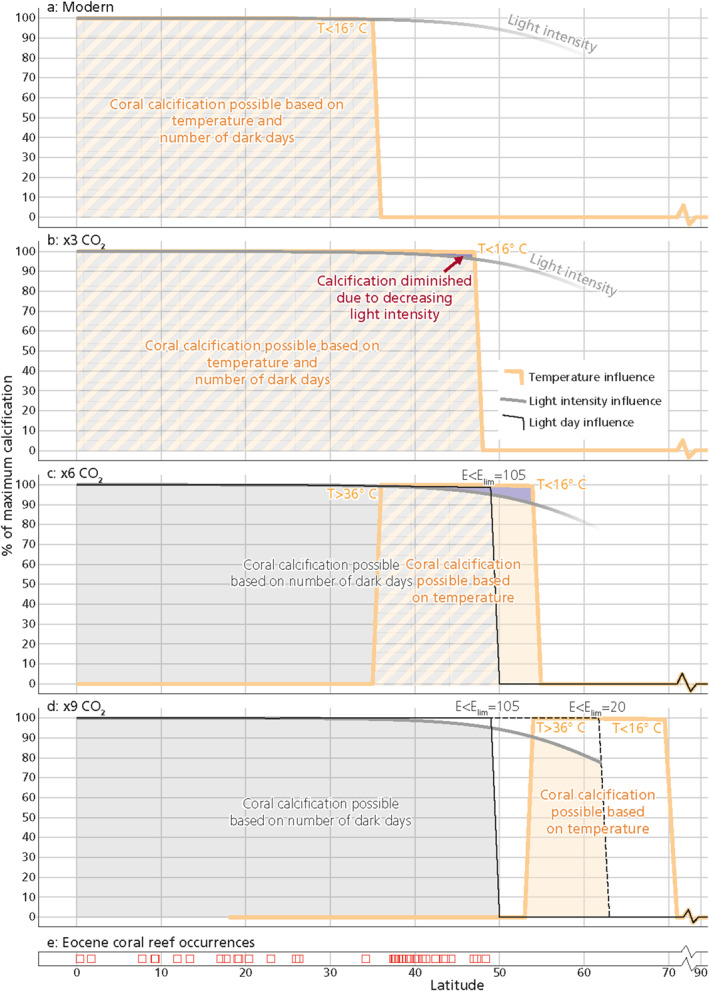
Yearly calcification rate at each latitude for the four different temperature scenarios panels (a–d), presented as percentage of the maximum yearly calcification that can be obtained for that scenario. The gray lines and shading indicate calcification potential as influenced by light intensity, black lines indicate the influence of dark days and yellow lines and shading indicate the calcification potential based on temperature influence. Beyond 40°, calcification potential drops due to temperature and light limitation. Between 0 and 35 (x6 simulation, panel c) or 53° latitude (x9 simulation, panel d), calcification potential is limited by high temperatures but not by light intensity or dark days. The light threshold (*E*_lim_) used to compute the number of dark days in each scenario was set to 105 μmol m^−2^ s^−1^, but note that the increase in number of dark days within one latitude differs per *E*_lim_ setting (Figure S3 in Supporting Information S1). Panel d also shows the dark day influence for a lower threshold of 20 μmol m^−2^ s^−1^ (dotted black line). Panel e shows the Eocene coral reef occurrences as obtained from the PARED database (Kiessling & Krause, 2022).

Our simulations reproduce temperature limitation of coral calcification in the modern ocean (Figure 2a). In the elevated‐temperature scenarios (x3, x6 and x9 CO_2_), they predict a decline in coral calcification rates starting at 40° latitude and a steepening in this decline from 50° onwards (Figures 2b–2d). This decline is controlled by light intensity, since Eocene winter temperatures at 40–50° latitude were >16°C and thus not limiting coral calcification in the model. Addition of a maximum tolerance to days without daylight leads to an abrupt cutoff in calcification rates beyond this threshold. Assumptions regarding *E*_*z*_ and *E*_*k*_ affect the shape of the decline in calcification rates (Figure S2 in Supporting Information S1). Lower *E*_*z*_, as varied through kpar, decreases the calcification rates (Figure S2a in Supporting Information S1). A higher minimum light‐requirement has the same effect on the model output; calcification rates diminish with higher *E*_*k*_ values (Figure S2b in Supporting Information S1).

In the modern and the lowest of the three elevated temperature scenarios (Figures 2a and 2b), the cut‐off in calcification potential is temperature controlled. In the Eocene x6 CO_2_ scenario, winter temperature would become limiting at 54°latitude and in the x9 CO_2_ scenario this would be at 71°latitude. We can only reproduce the Eocene coral data by setting *E*_lim_ to at least 105 μmol m^−2^ s^−1^ with a darkness tolerance of 1–26 days (Figures 2c and 2d and Eocene coral reef record in 2e), restricting coral reefs to >50° before winter temperatures fall below 16°C in both the x6 and the x9 CO_2_ scenario. With a lower *E*_lim_ of 20 μmol m^−2^ s^−1^, light availability determines the poleward limit of coral occurrence only in the x9 CO_2_ simulation (dotted black line in Figure 2d) and does not yet explain the lack of coral reef occurrences >50° in the geological record of the Eocene (Figure 2e). Figure S3 in Supporting Information S1 shows the yearly number of dark days at each latitude for all tested values of *E*_lim_. The other way to approach the Eocene coral distribution is to assume a significantly higher minimum temperature than the current limit of 16°C.

In the x6 and x9 CO_2_ scenarios, calcification potential is limited by high temperatures at low latitudes (<35° or <53°, respectively), where summer temperatures rise above 36°C (yellow area in Figures 2c and 2d indicating the thermal niche). In fact, the modeled thermal niche for corals in the x9 CO_2_ scenario lies completely beyond latitudes in which corals were found in Eocene sediments. A decline in low latitude coral‐reef production is indeed recorded in the geological record of the hottest periods of the Eocene (Perrin & Kiessling, 2010; Scheibner & Speijer, 2008), but corals still colonized regions close to the equator (Zamagni et al., 2012). The discrepancy between our modeled thermal niche and the coral record leads us to hypothesize that corals tolerated much higher temperatures during the Eocene than corals do today. This has also been suggested by Zamagni et al. (2012), who proposed that coral adaptation might have occurred through natural selection for more heat‐tolerant lineages of corals or more heat‐tolerant lineages of the algal endosymbiont and points at geological evidence in the coral record for at least the first of the two adaptation mechanisms.

## 4 Discussion

### 4.1 Tolerance to Darkness

Multiple factors govern the distribution of present‐day corals, but temperature, light availability and aragonite saturation state are the most important (Kleypass et al., 1999). Traditionally aragonite and temperature received most attention, but light availability sets the energy supply (from phototrophic symbionts) and thus is the ultimate controlling factor. Moreover, reconstructions of carbonate ion conditions showed that the ocean remained aragonite saturated over the Cenozoic (Boudreau et al., 2019). It is for this reason that we focus on temperature and light as controlling factors. Our results imply that light intensity and day‐length should be considered in predictions of past and future coral migration and growth. We also showed that the hypothetical tolerance of a coral to a fixed number of days spent in the dark has a large impact on the latitudinal extent of their occurrence. At present, tropical corals receive daylight year‐round and no clear understanding of this tolerance of tropical corals to longer periods of darkness exists (Bessell‐Browne et al., 2017). This relationship between darkness and coral survival is hard to study experimentally or in the field, since the relatively short timeframes of such studies do not allow for adaptation and evolution to changing light regimes that might occur in a natural setting. Studies on corals growing at their environmental limits have shown that the ability of coral species to acclimatize to the prevailing light conditions, has a big influence on their abundance and distribution (Sommer et al., 2018). However, the lack of coral reefs in the geological record of the Eocene above 50° (Figure 1; PARED database, Kiessling & Krause, 2022) suggests that tropical corals here did not have sufficient capacity to adapt. We therefore hypothesize that reef forming corals cannot survive longer stretches of darkness. In our model, the threshold light value below which a day is considered “dark” is set by parameter *E*_lim_. This parameter is however not well established. Many studies have assumed a general reef‐limiting level of 10% of “surface light”, but this is problematic since surface light varies with location. Kleypas (1997) found that a value of 250–300 μmol m^−2^ s^−1^ (11–13 mol m^−2^ day^−1^, for a 12 hr day) best reflects present‐day global reef area estimates. Gattuso et al. (2006), however, deemed this a conservative estimate of limiting light level, since it reflects the light level below which CaCO_3_ accumulation is no longer positive, but does not consider the level at which gross primary production exceeds carbon losses. Coral communities can still contribute to net primary production while no longer producing an excess of CaCO_3_. They suggested a reef‐limiting light level of 1.5 mol m^−2 ^day^−1^ (35 μmol m^−2^ s^−1^, for a 12‐hr day).

By fitting our model parameters *E*_lim_ and the dark‐day tolerance to the observed drop in coral occurrence beyond 50°, we find that an *E*_lim_ of at least 105 μmol m^−2^ s^−1^ is needed to explain the fossil record assuming a minimum growth temperature of 16°C (Figures 2c–2e, Figure S3 in Supporting Information S1). Higher values of *E*_lim_ in combination with a longer tolerance to dark days produces the same result (Figure S3 in Supporting Information S1). Our study can thus only provide hypothetical combinations for these two parameters.

### 4.2 Implications

In the warm climate of the Eocene, the lack of coral reefs at high latitudes can be explained more satisfactorily by decreased light levels than critically low temperatures (Figure 2). This challenges the premise (e.g., Davies et al., 2019) that the absence of coral reefs implies that mixed‐layer water temperatures were below 16°C at latitudes >50°. Moreover, in carbon cycle studies, climate warming is typically assumed to cause latitudinal expansion of carbonate reefs and therefore marginal marine alkalinity burial (e.g., Opdyke & Wilkinson, 1993; Sluijs et al., 2013), but our work shows that this assumption is problematic under warm climate states.

Our study has focused on comparing coral reef distributions from the Eocene with present day warm‐water coral distributions. The fundamental principle that adequate light intensity and duration are crucial for the survival of warm‐water corals can be extended to other past climates, notably those where temperatures at mid to high latitudes were not limiting for coral growth, such as the Cretaceous (O’Brien et al., 2017).

Significant light limitation requires temperatures of at least 16°C reaching mid and high latitudes; ∼10°C warmer than today. Current global warming projections for the coming century are on the order of 3°C (IPCC, 2021) so we do not expect light limitation to play an important role in near‐future poleward tropical coral reef expansion.

Further work is required to see whether light availability might have played a pivotal role in constraining the latitudinal patterns for Triassic, Permian and Paleozoic reefs (Martindale et al., 2015, 2019) or during the protracted recovery of reefs following the K‐Pg event due to global darkness (Vellekoop et al., 2014). However, non‐analog reefs in the past may have had different light requirements or even been non‐photosymbiotic, as present‐day cold‐water corals.

## 5 Conclusions

Our model simulations imply that while temperature at present controls the latitudinal extent of coral reef habitats, daily irradiance, the energy supplier to corals, determined the uppermost latitudinal limit during past warm climates. Temperature did not limit coral growth at high latitudes in the Eocene, but light limited their extent to ∼50°N. Since coral tolerance to darkness is not well established, our model simulations provide a hypothetical limitation to coral reef occurrence based on their past latitudinal extent. For the correct interpretation of coral reef records from hothouse climates of the past and for the predictions of latitudinal migration potential of coral reefs under future hothouse climates, light availability and a corals relationship with light needs to be taken into consideration.

## Supporting information

Supporting Information S1

## Data Availability

All model code and temperature data used for the simulations can be found on https://github.com/AnneKruijt/Coral_Light_modelcode and are preserved on Zenodo (Kruijt, 2024). Eocene temperature data used in the simulations were downloaded from Lunt et al. (2021), who refer to the CESM simulations by Zhu et al. (2019). The Eocene reef occurrences as plotted in Figure 1 were downloaded from the PARED database: https://www.paleo‐reefs.pal.uni‐erlangen.de (Kiessling & Krause, 2022). The downloaded data and a description of the search terms used in the database can be found in the github folder. Modern day temperature data were obtained from the NOAA atlas (Locarnini et al., 2019). The R‐package oceanexplorer (Schobben, M., 2023) was used to download the temperature files from the Ocean Atlas. Both the Eocene and the modern day temperature data have been made available on github by AK, in the correct format for performing the simulations in this article.
