# Supplementary material for: Light Limitation of Poleward Coral Reef Expansion During Past Warm Climates
Source: Geophys Res Lett. 2024 Nov 21;51(22):e2024GL111757. doi: 10.1029/2024GL111757 (PMC11579961; doi:10.1029/2024GL111757)
Supplement: Supplementary file 1 — Supporting Information S1 [file GRL-51-0-s001.pdf]

[Geophysical Research Letters]

Supporting Information for

Light limitation of poleward coral reef expansion during past warm climates

A. L. Kruijt1, T. Brachert2, A. Sluijs,1 J. J. Middelburg1

1 Department of Earth Sciences, Faculty of Geosciences, Utrecht University, Princetonlaan 8A, 3584CS Utrecht, The Netherlands

2 Institute for Earth System Science and Remote Sensing, University of Leipzig, Talstrasse 35, 04103 Leipzig, Germany

Contents of this file

- Text S1: Model equations for the light regime
- Figures S1 to S3

Introduction

This file contains a description of the equations used to model the light regime in the simulations described in the article. It also contains three supporting figures showing 1) the modeled irradiance curves used for the light regime, 2) the results of sensitivity tests for E_k and kpar and 3) the relationship between latitude, E_{lim} and the number of dark days.

Text S1.

Model equations for the light regime

Irradiance calculations

Daily irradiance at the top of the atmosphere for a specific location on earth is a function of the solar declination angle (d, angle between the sun and the Earth's equatorial plane) for this specific day (DOY), the sun hour angle (ω_s) and the latitude (lat) of this location. In this study we adopt the functions for daily irradiance as described by Berger (1978) and Duffie and Beckman (2013):

$$E_{daily} = E_{corr} * I_{sc} * (\omega_s * \sin(lat) * \sin(d) + \cos(lat) * \cos(d) * \sin(\omega_s)) \text{ (S1)}$$

We assume irradiance levels from the sun were constant over the domain of our study and set I_{sc} to 1367 Watt m^{-2}. The correction factor E_{corr} is used to account for the change in distance between sun and the earth throughout the year:

$$Ecorr = 1 + 0.0033 * \cos\left(\frac{2*\pi}{365} * DOY\right) \quad (S2)$$

The declination angle (in degrees) is computed as follows:

$$d = 23.45 * \sin\left(2\pi * \frac{(284+DOY)}{365}\right) \text{ (S3)}$$

The sun hour angle (ω_s) is calculated based on the latitude and the declination. If the absolute value of the angle between the latitude minus the declination angle is larger than 90°, this means the sun never rises and the sun hour angle is 0. If the absolute value of the angle between the latitude plus the declination angle is larger than 90°, this means the sun never sets and the sun hour angle is 180° or π radians. In all other cases, the sun hour angle is calculated as follows (result in radians):

$$\omega_s = \text{acos}(-tan(lat) * tan(d)) \quad \text{(S4)}$$

Scaling of irradiance to surface ocean observations

The above equations allow us to account for the change in radiation with time of year and latitude. The amount of light reaching the earth's surface further depends on atmospheric processes. We choose not to parameterize or model these explicitly. Instead we assume a fixed maximum irradiance value of 650 μmol m^{-2} s^{-1} at the earth surface and scale the irradiance curves to this maximum value:

$$E_{daily\_scaled} = \frac{1}{(E_{daily\_max})} * E_{daily} * E_{obs\_max} \quad \text{(S5)}$$

This maximum value is based on the assumption that average daily irradiance at the equator is 500 μmol m^{-2} s^{-1} (Kleypas, 1997; Pinker and Lazlo, 1992). We fit this to our modeled irradiance curves, resulting in a maximum obtained irradiance at the earth's surface of 650 μmol m^{-2} s^{-1}, occurring at the poles in summer months.

Refraction at air-water interface

The amount of light penetrating through the water at a given day and latitude further depends on the refraction at the at the air-water interface. The angle of the refracted beam is calculated with Snell's law:

$$Phi_w = \text{asin}\left(na * \frac{\sin(Phi_a)}{nw}\right) \quad \text{(S6)}$$

With the index of refraction of air, na, set to 1 and the index of refraction of water, nw, set to 1.335.

Phi_a is the acute angle between the incident light beam and the normal to the surface of the water and calculated by subtracting the earths declination from the latitude of interest (for the angle at noon):

$$Phi_a = lat - d \quad \text{(S7)}$$

The fraction of light that is reflected is calculated with Fresnels equations:

$$R_{perp} = \frac{\sin^2(Phi_a - Phi_w)}{\sin^2(Phi_a + Phi_w)}$$
$$R_{parallel} = \frac{\tan^2(Phi_a - Phi_w)}{\tan^2(Phi_a - Phi_w)}$$
$$R_{effective} = 0.5 * (R_{perp} + R_{parallel}) \quad \text{(S8a,b,c)}$$

The amount of light that enters the water surface is then corrected for the effective reflection at the air-water interface:

$$E_{surface} = E_{daily\_scaled} - E_{daily\_scaled} * R_{effective} \quad \text{(S9)}$$

The effect of wave action on the light penetration is not taken into account in this study

Supporting figure S1: Irradiance curves

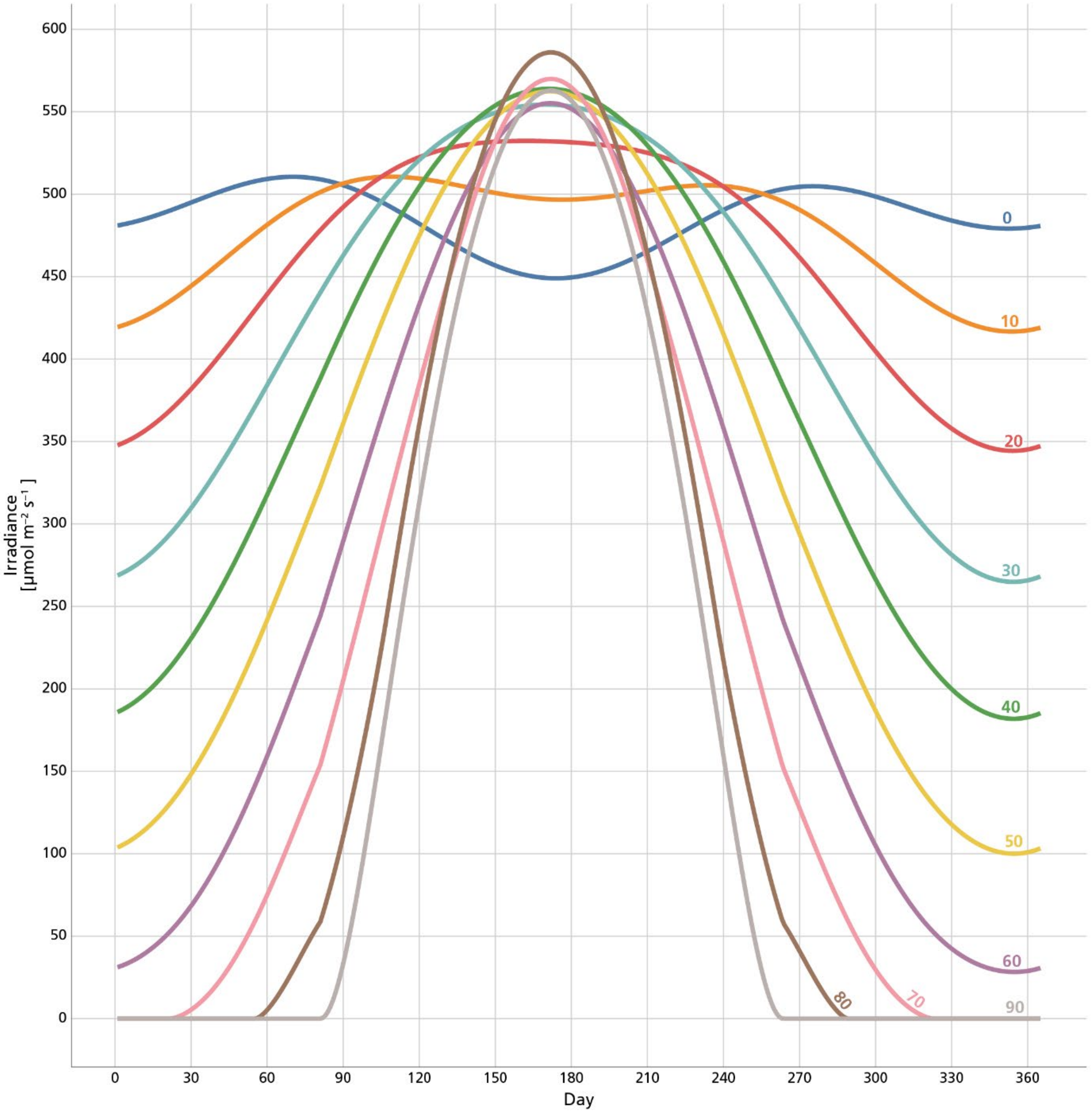

Figure S1: Light entering the water column ($E_{surface}$) for each day of the year at latitudes 0-90°, as indicated by the colored lines.

Supporting figure S2: Sensitivity test of kpar and E_k

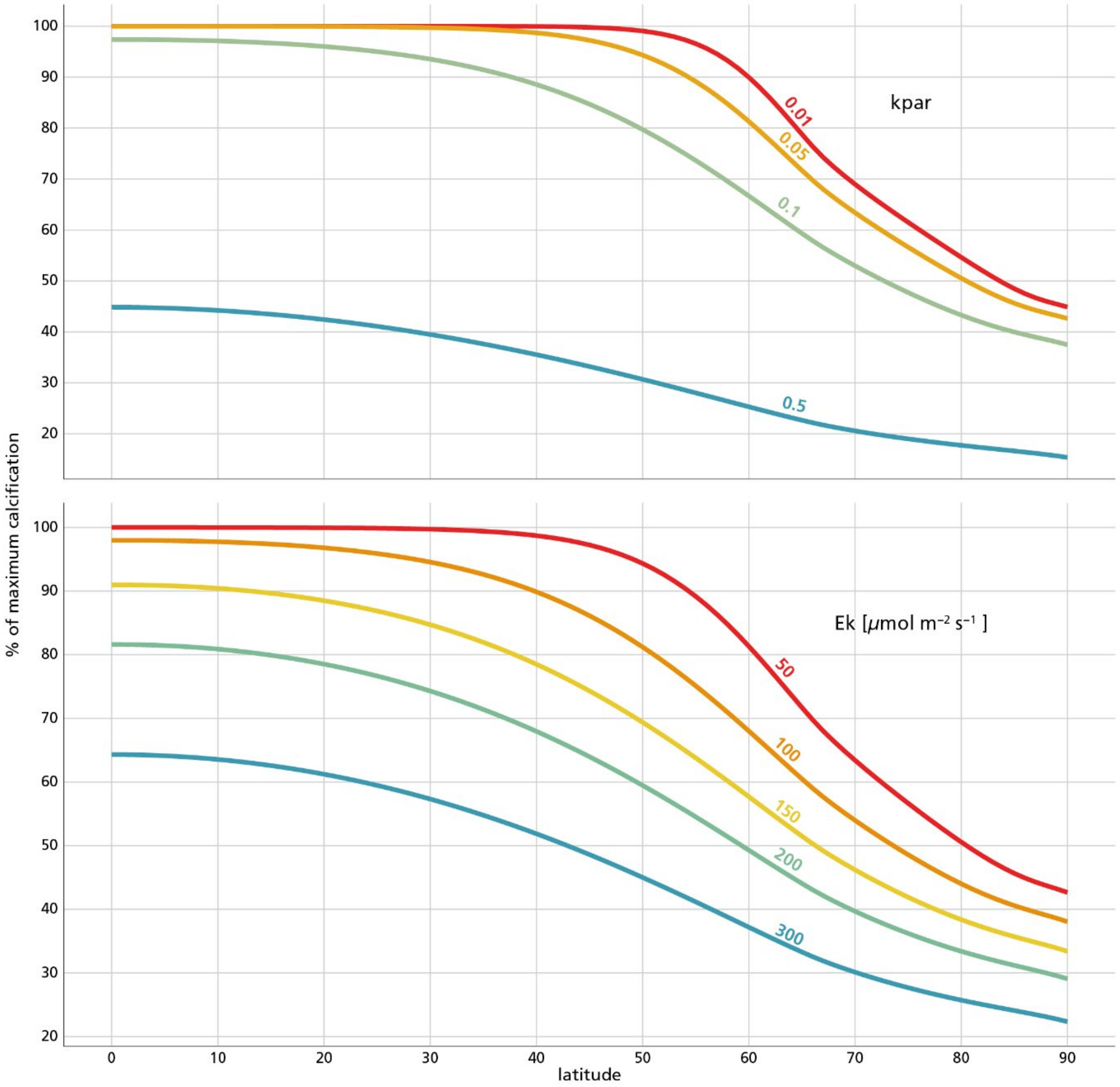

Figure S2: Potential calcification with latitude is plotted for a range of kpar (A) and E_k (B) values. Temperature is assumed unlimiting in these simulations, in order to show the sole effect of changing light availability on the calcification rates.

Supporting figure S3: Effect of E_{lim} on relationship between latitude and dark days

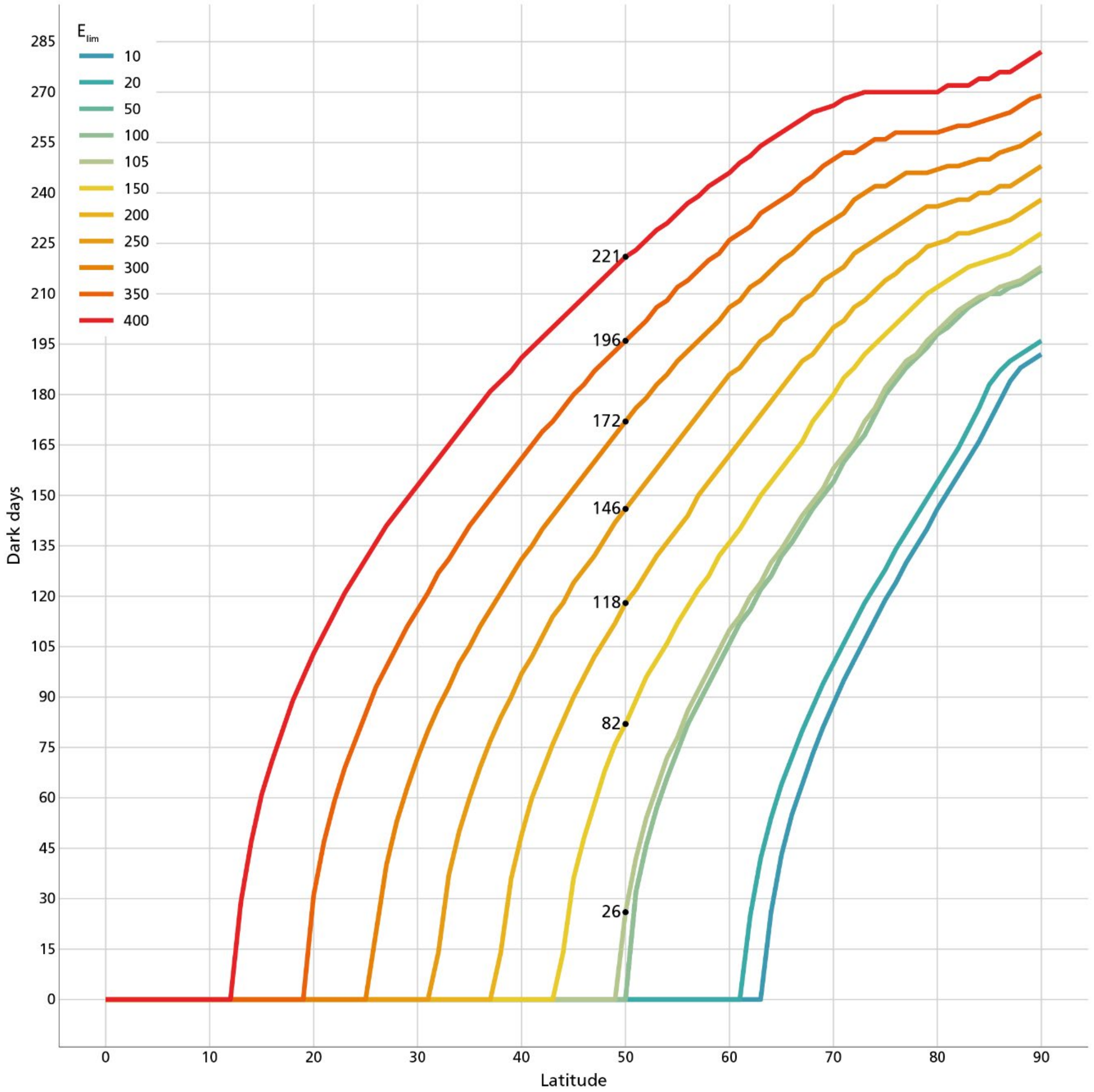

Figure S3: Yearly number of dark days occurring at each latitude, for different values of E_{lim}. The rate at which the number of dark days increases with every degree latitude differs per value of E_{lim}. The black dots indicate what the tolerance to dark days must be for the latitudinal limit to calcification to be reached at 50°, for each value of E_{lim} .
